# Physiological Response to Cycling With Variable Versus Constant Power Output

**DOI:** 10.3389/fphys.2020.01098

**Published:** 2020-08-27

**Authors:** Erik Borg Kolsung, Gertjan Ettema, Knut Skovereng

**Affiliations:** Centre for Elite Sports Research, Department of Neuromedicine and Movement Science, Norwegian University of Science and Technology, Trondheim, Norway

**Keywords:** cycling, performance, variable power, constant power, physiological response

## Abstract

**Introduction:** Variable power output (VP) is one of the main characteristics of a road cycling mass-start. Tolerating VP during outdoor road cycling highly influences performance. There is a lack of continuous and comprehensive measurements during this power condition. Accordingly, the aim of the present study was to investigate physiological response to VP vs. constant power output (CP) as well as the perceived exertion of these two power conditions, and to investigate if variations in power output which span above lactate threshold (LT), differ from variations below LT.

**Methods:** 15 elite competitive cyclists completed three test days, including 1 day of baseline testing and 2 days of main testing, consisting of four bouts of 28 min at two different intensities, “low” at 70% of LT and “high” at 95% of LT, with VP and CP. VP was performed with a 15% fluctuation of the average power output every second minute. Maximal oxygen uptake (VO_2_), respiratory exchange ratio (RER), heart rate (HR), blood lactate (LA), rating of perceived exertion (RPE), cadence (RPM) and power output (W) were measured.

**Results:** At both low and high intensity, the VP condition induced a significantly higher VO_2_, HR and LA than the CP condition. Whole-bout RPE was similar between power conditions at high intensity. Additionally, at the high intensity, cycling with VP led to a greater increase in LA and lesser increase in RPE compared to cycling with CP.

**Discussion:** The results of this study show that, despite considerable differences in the demand during the VP and CP bouts, there are minor differences in the perceptual and physiological response directly following these two power conditions in a cohort of elite competitive cyclists. A practical implication of these findings is that training with VP seems to be a viable alternative to training with CP, at least at high intensity.

## Introduction

One of the main characteristics of competitive cycling is variations in power output (Faria et al., 2005; Ebert et al., 2006) which occur as a consequence of changing weather conditions, changing terrain and the group dynamics of the peloton (e.g., drafting) (Palmer et al., 1994). To perform well and to win races, cyclists need to be able to tolerate variations in power output. The effects of a variable power output (VP) versus constant power output (CP) have previously been studied, both in terms of pacing strategies (Atkinson and Brunskill, 2000), subsequent performance (Palmer et al., 1997), and isolated physiological response (Liedl et al., 1999). In a time-trial, a pacing strategy with constant velocity produces the best performances (Foster et al., 1993; Swain, 1997; Atkinson and Brunskill, 2000), meaning that a CP strategy is superior when the course is completely flat and without wind (e.g., track cycling). In contrast, mathematical models have demonstrated that increasing power output with as little as 5% when riding uphill or into headwind, and a corresponding decrease when riding downhill or in tailwind, will lead to a better time-trial performance than keeping the power output constant (Swain, 1997; Wells and Marwood, 2016).

Training with CP is common among cyclists and other endurance athletes. Traditional interval training during the preparation period often consist of longer work bouts (∼8–15 min) with CP, with a work rate at or slightly below the lactate threshold (LT) (Solli et al., 2017; Rønnestad and Hansen, 2018). Considering the training principle of specificity, training with VP may, in many cases, mimic race situations to a greater extent than training with CP. Even though designing training programs concerns more than just the principle of specificity, it could be argued that cyclists advantageously could include more VP work into their training. However, if the physiological cost of VP differs from CP, careful considerations regarding total training load and periodization of these workouts would be necessary when designing training programs.

Liedl et al. (1999) reported that no additional physiological cost in terms of VO_2_, heart rate and lactate was found after a 1-h effort performed with VP compared to the same effort performed with CP. Similarly, Brickley et al. (2007) reported that variations in power output did not have a significant effect on muscle metabolism. However, elevated blood lactate (LA) levels during VP compared to CP has previously been reported (Palmer et al., 1999; Suriano et al., 2007) and greater power variation have been shown to increase neuromuscular fatigue (Theurel and Lepers, 2008).

Although the knowledge is growing on the physiological response to VP and CP, there is a lack of continuous and comprehensive measurements of performance related variables [e.g., LA, oxygen consumption (VO_2_), metabolic rate (MR) etc.] during VP and CP. Previous studies have neither investigated physiological response in competitive elite cyclists, which may be more accustomed to variations in power output from their racing experience, nor looked at VP and CP differences when performing low intensity exercise, which cyclists do to a large extent.

The training intensity distribution of elite endurance athletes, including cyclists, is approximately 80% low-intensity (LIT) and 20% moderate- (MIT) and high-intensity (HIT) (Seiler and Tønnesen, 2009; Seiler, 2010). Additionally, as much as 40–45% of flat and hilly mass-start stages of the Tour Down Under (Ebert et al., 2006) was spent at an intensity that could be classified as LIT (i.e., <1.9 W/kg). While the power output during the large LIT volume in training can be controlled and quite constant, the power output corresponding to LIT during races would include more variation due to the previously mentioned peloton dynamics. However, if CP and VP during LIT differs in physiological and perceptual cost has, to the best of our knowledge, not been investigated.

Accordingly, the primary aim of the present study was to investigate physiological and perceptual response to VP vs. CP in elite competitive cyclists. The secondary aim was to investigate if variations in power output which span above LT, differ from variations below LT. Based on the exponential nature of LA accumulation above LT (Faude et al., 2009), the substantial change in VO_2_ kinetics above LT, including the slow component (Lucìa et al., 2000), and the findings of previous studies with their respective protocols, the hypothesis of the present study was that cycling with a VP condition would induce higher mean physiological cost in terms of VO_2_, HR, and LA as well as a higher RPE, than a CP condition, but only at the high intensity where the power variation fluctuated above and below LT.

## Materials and Methods

### Participants

19 elite competitive male cyclists (Table 1) were recruited through local clubs and teams in the area of Trondheim, Norway. Four of the participants were excluded – three of them due to measurement errors and one of them because he was not able to complete the full protocol. The study was conducted in the preparation period (i.e., November to January) for elite competitive cyclists. All of the participants were experienced with bike racing and seven of the participants had competed in races categorized by the Union Cycliste Internationale (UCI) as category 1.2 or higher. The participants were informed verbally about the procedures and purpose of the study and signed an informed written consent prior to participation. When explaining the study, we only stated that we wanted to compare VP and CP at high and low intensity, and we made sure not to mention our hypothesis about the study. The study was performed according to the Helsinki Declaration of 1975 and was registered and approved by Norwegian Social Science Data Services.

**TABLE 1:** Characteristics of the 15 included cyclists (mean ± SD).

Age (years)	24.9 ± 7.6
Weight (kg)	72.6 ± 7.3
Height (cm)	182.2 ± 7.1
VO_2max_ (ml/kg/min)	72.9 ± 5.1
VO_2max_ (L/min)	5.3 ± 0.4
LT (W)	310.5 ± 21.7
LT (W/kg)	4.3 ± 0.4
PPO (W)	415.0 ± 28.0
PPO (W/kg)	5.8 ± 0.5
Number of races last season	33.9 ± 17.2
Training volume last season (hours)	691.0 ± 186.6

*LT, lactate threshold; PPO, peak power output; VO_2max_, maximal oxygen uptake.*

### Experimental Protocol

Each participant came to the laboratory on three occasions at a standardized time of day, each preceded by at least 1 day of low intensity, short-duration exercise. Each participant completed the three test days within 6 ± 4 days.

The first day of testing was baseline testing consisting of a lactate profile test and VO_2__max_ test (Sylta et al., 2016). After the measurement of height and body mass, the participants were allowed a 10-min warm-up with freely chosen cadence at an intensity <70% of LT before the lactate profile test started at a power output of 125 W with 50 W increases every 5 min. After reaching a LA of 2 mmol/l, work rate increments were reduced to 25 W and the test was terminated when LA exceeded 4 mmol/l. A lactate sample was taken at the end of every step and the power output corresponding to 4 mmol/l was determined as LT. The protocol that we used to determine power output at 4 mmol/l is a known protocol for our participants, with many of them having performed it several times each year during the past years. Having the 4 mmol/l protocol in our study, we also gave something back to the participants, i.e., they used the results of the first test day in their own training prescription/evaluation. The protocol has also previously been used in other studies (Sylta et al., 2016).

Following a 10-min active recovery with a freely chosen cadence and an intensity <70% of LT, a VO_2__max_ test was performed. No specific measurements of recovery from the lactate profile test was taken. The VO_2__max_ test started at the same load as the penultimate stage from the lactate profile test, and work rate was increased by 25 W every minute until volitional exhaustion while given strong verbal encouragement. Peak power output was calculated as the mean work rate during the final minute of the test and VO_2__max_ was calculated as the highest 1-min average during the test. VO_2_, heart rate and cadence were measured continuously, and blood lactate was measured 1 min after the completion of the test. Considering that the participants in this study were elite and that they were familiar with the lactate profile test and the VO_2__max_ test in a combined protocol, we are confident that the recovery time between the two tests was sufficient.

**FIGURE 1:**
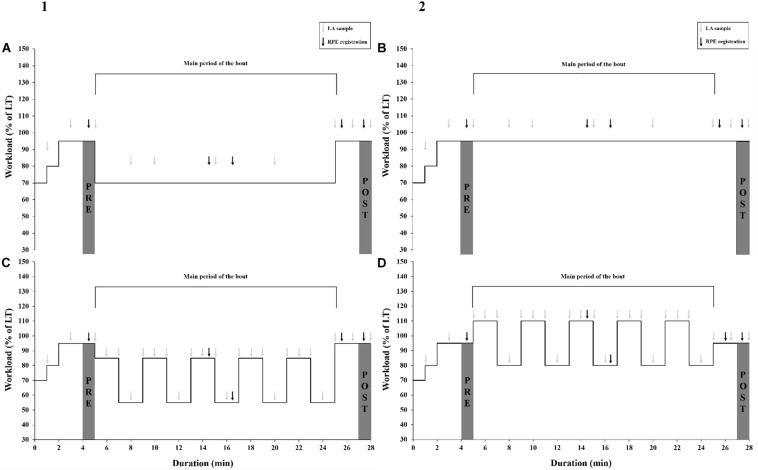
The protocol and design of the four bouts in the present study, **(A)** low constant (LC), **(B)** low variable (LV), **(C)** high constant (HC), **(D)** high variable (HV), with the incremental ramp of 5 min, the main period of the bout with work-matched VP or CP, and the final 3 min of constant power. Oxygen consumption, respiratory exchange ratio, heart rate, power output and cadence were continuously measured throughout the bout. Gray arrows indicate the time points that LA concentration was measured at. Black arrows indicate the time points that rating of perceived exertion was registered at. The gray bars visualize the time periods used for the PRE (mean during the 5th min for oxygen consumption and heart rate, lactate at the end of the 5th min and rating of perceived exertion registered after 4.5 min) and POST (mean during the 28th min for oxygen consumption and heart rate, lactate at the end of the 28th min and rating of perceived exertion registered after 27.5 min) comparison.

The second and third day of testing started with a 25-min warm-up at low intensity (<70% of LT) with freely chosen cadence, followed by a low intensity bout and a high intensity bout with VP or CP, separated by 10 min of active recovery (freely chosen cadence and <70% of LT) (Figure 1). All bouts included an identical 5 min incremental ramp, a 20 min main period with work matched CP or VP, and a final 3 min at 95% of LT. The 3 min at 95% of LT prior to and after each of the four conditions were done in order to compare the effects of the different bouts at a standardized load and was not meant to directly reflect the high and low conditions, respectively. The high and low conditions were performed at an average power output of 95% and 70% of LT, respectively, and the VP condition was performed with a 15% fluctuation every second minute. This 15% amplitude of fluctuation allowed the highest intensity to reach 110% of LT and thus achieve our aim of a condition above steady state. The 2 min duration of fluctuations was chosen in order to allow for changes in physiological variables to occur, while not being so long that the highest intensity would be exhaustive.

All bouts were performed with a freely chosen cadence and in a seated position. The cyclists were allowed only short periods of standing (<10 s) for comfort reasons. The time points for the LA and RPE measurements are shown in Figure 1. RPE was measured using Borg’s 6–20 RPE scale and the participants were instructed to rate their whole-body feeling of exertion at the current time of measurement. All participants were familiar with the Borg’s scale both from prior use and from the first day prior to the second and third day of testing. Whole-bout RPE-score was measured straight after the final 3 min at 95% of LT in each bout and the participants were instructed to rate their level of exertion for the bout as a whole.

The low intensity bout was performed before the high intensity bout, due to the expectation of a lesser influence from the low intensity bout on the high intensity bout than the opposite order. The order of power strategy (VP or CP) was randomized, which mean that the participants were randomized into 4 possible orders, i.e., low variable (LV) or low constant (LC) followed by high variable (HV) or high constant (HC) on day 1, and then the remaining two bouts on day 2. The distribution across the 4 possible randomization orders for our 15 included participants was 3, 4, 5, and 3 athletes, respectively. Food and drinks were allowed in between the two bouts. The participants were instructed to replicate quantity and type of food and drink intake from day 2 to day 3 of testing.

### Equipment and Measurements

All testing was performed in a laboratory with stable conditions (temperature ∼18°C and relative humidity ∼30%). The cyclists used their own road bike on an electronically braked indoor cycle trainer (CompuTrainerTM, RacerMate^®^ Inc., Seattle, United States) calibrated according to the manufacturer’s instruction and using a standardized tire pressure of 7 bar. An electrical fan was made available during all tests. A 20 μl blood sample was collected from the participant’s fingertip and LA was analyzed using the Biosen C-Line Sport lactate measurement system (EKF Industrial Electronics, Magdeburg, Germany). HR was measured with a heart rate monitor (Garmin Forerunner 920XT, Garmin International Inc., Kansas, United States). VO_2_ and RER were measured with an open-circuit indirect calorimetry apparatus (Oxycon Pro, Jaeger GmbH, Hoechberg, Germany). The apparatus was calibrated on every test day, using a gas of known concentration (15.0% O_2_ and 5.0% CO_2_, Riessner-Gase GmbH & Co., Lichtenfels, Germany) and a 3-liter syringe (Hans Rudolph Inc., Kansas City, MO, United States). VO_2_, RER, HR, RPM, and W were continuously measured throughout all the four bouts during main testing.

### Data Analysis

Mean VO_2_, RER, HR, and LA were calculated for the whole 20-min main period and for the last minute average for every high and low power output segment during the 20 min period of VP and the corresponding segments of CP (e.g., the first upper power segment of the VP bout was from 05:00 to 07:00, so the time 06:00–07:00 was used in the VO_2_, RER and HR calculation for both the VP and CP). We expected more variations in LA during VP compared to CP (Beneke, 2003), thus, LA was sampled more frequently during the VP than CP. The LA data for the CP bouts were interpolated in order to compare the data.

To investigate the effect of the whole 20-min main period, mean VO_2_, RER, and HR was calculated for the last min prior to the main period, as well as for the last min of the bout (see gray bars representing the PRE and POST periods in Figure 1). RPE for the PRE and POST periods was measured after 4.5 and 27.5 min and LA was measured at the end of the PRE and POST periods.

Metabolic rate (MR) was calculated from VO_2_ and RER measurements, converted to energy expenditure (Peronnet and Massicotte, 1991).

### Statistical Analysis

All descriptive data are presented as mean ± standard deviation. Raw data were visually inspected to check for possible measurement errors prior to further analysis. The main analysis was done using a two-way repeated measures ANOVA to investigate main effects of intensity and power condition as well as to check for possible interaction effects on VO_2_, LA, HR, and RPE. When comparing the PRE to POST values, the delta values were calculated and used in the two-way ANOVA. Where significant effects were found, contrast analysis and pairwise comparisons using Bonferroni correction was used to determine specific effects of intensity and power condition. Strength of the associations in the two-way ANOVA was evaluated using partial eta squared (η^2^).

Additionally, a paired samples *t*-test was used for comparing values at PRE and POST in terms of VO_2_, LA, HR, RER, and RPE as well as for investigating segment differences between the two power conditions for both intensities. Statistical significance was accepted at *p* < 0.05 and where Bonferroni correction was applied, the corrected alpha is stated. All statistical analyses were conducted using SPSS 25.0 (SPSS, Chicago, United States) for Windows.

## Results

**FIGURE 2:**
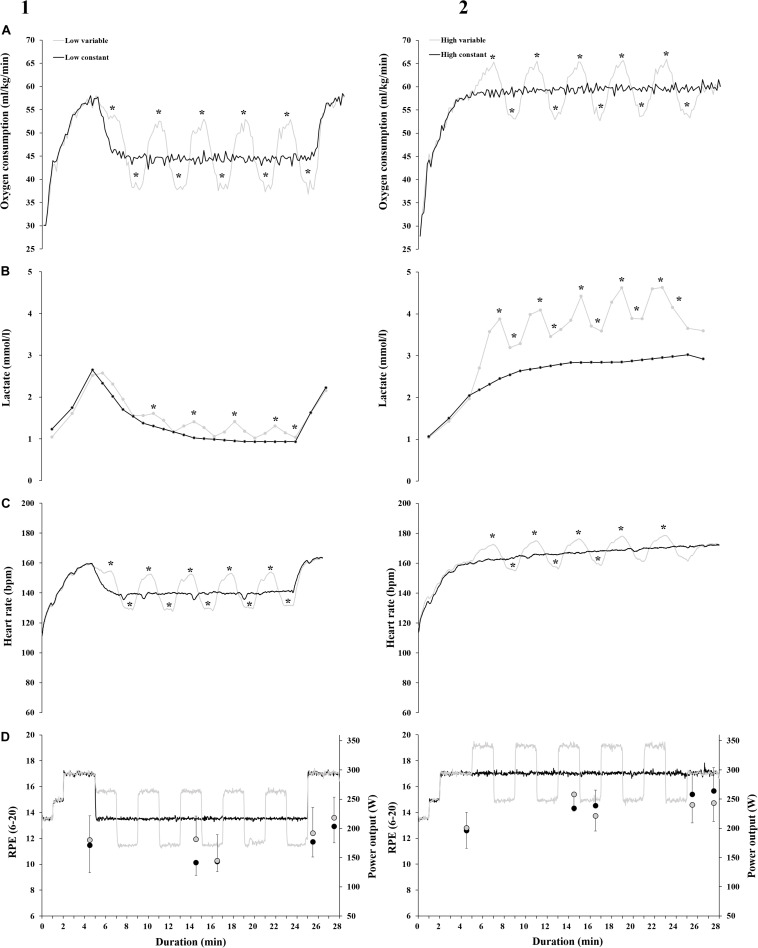
Mean **(A)** oxygen consumption (VO_2_), **(B)** blood lactate (LA), **(C)** heart rate (HR), and **(D)** power output (W) and rating of perceived exertion (RPE) during variable power (gray line) and constant power (black line) at (1) low intensity and (2) high intensity. Gray circles refer to RPE for VP, black circles refer to RPE for CP. The LA data plotted for the two constant power bouts are interpolated, as specified in the methods. * indicate a significant difference in VO_2_, LA or HR during the variable power segment compared to the corresponding constant power segment, *p* < 0.05.

The VO_2_, LA, and HR response during the bouts were significantly affected by power condition and intensity (Figure 2 and Table 2). There was no significant difference in average power output of the 20 min main period of the bout between LV (218 ± 15 W) and LC (217 ± 15 W) (*p* = 0.43), nor between HV (294 ± 20 W) and HC (294 ± 20 W) (*p* = 0.28). Cadence was not different across the four bouts (HC: 87.5 ± 7.2, HV: 88.2 ± 5.0, LC: 87.2 ± 5.5, LV: 87.8 ± 4.9) nor in any PRE to POST comparison (all Δ < 2.2 rpm and all *p* > 0.26). Mean VO_2_ of the bouts corresponded to 63, 62, 82, and 81% of the participants VO_2__max_, for LV, LC, HV, and HC, respectively.

**TABLE 2:** Mean ± standard deviation for different variables during the 20 min main period of the bout for all four bouts.

	LC	LV	HC	HV	Cp	Cη^2^	Ip	Iη^2^	Intp	Intη^2^
VO_2_ (ml/kg/min)	44.9 ± 3.4	46.0 ± 3.7*	59.2 ± 4.3	59.9 ± 4.1	<0.05	0.521	<0.01	0.990	0.33	0.068
LA (mmol/l)	1.2 ± 0.3	1.4 ± 0.4*	2.8 ± 0.8	3.9 ± 0.7*	<0.01	0.832	<0.01	0.970	<0.01	0.668
Heart rate (bpm)	138 ± 3.2	139 ± 9.4	167 ± 3.0	168 ± 6.5	0.24	0.099	<0.01	0.973	0.43	0.046
RPE (6–20)^1^	11.3 ± 0.7	12.3 ± 1.6*	14.9 ± 1.3	14.9 ± 1.4	0.09	0.192	<0.01	0.940	0.09	0.194
RER (-)	0.90 ± 0.03	0.90 ± 0.04	0.91 ± 0.03	0.91 ± 0.05	0.68	0.012	<0.01	0.770	0.08	0.205
MR (W)	1153 ± 81	1174 ± 79*	1508 ± 94	1520 ± 104	<0.05	0.377	<0.01	0.990	0.47	0.038

Oxygen consumption (VO_2_), Blood lactate (LA), Heart rate, Rating of perceived excertion (RPE), Respiratory exchange ratio (RER) and Metabolic rate (MR) for each of the four bouts [i.e., low constant (LC), low variable (LV), high constant (HC) and high variable (HV)]. *P*-values and effect size (partial Eta squared) corresponding to main effects of power condition (Cp and Cη^2^), intensity (Ip and Iη^2^) and interaction effects (Intp and Intη^2^). ^1^RPE refers to whole bout. *indicates a significant difference at VP compared to CP at the corresponding intensity evaluated by a *t*-test, *p* < 0.05.

The differences between the upper and lower power segments of VP and the corresponding segments of CP are visualized in Figure 2. During HV, all segments had a significantly higher LA compared to HC (*p* < 0.002). 8 of 10 upper and lower power segments for HR, and 10 of 10 segments for VO_2_ had significantly higher and lower VO_2_ and HR, respectively, during HV compared to the corresponding segments of HC (*p* < 0.005). Every upper and lower power segment during LV had higher and lower VO_2_ and HR, respectively, compared to corresponding LC-segments (*p* < 0.005). At low intensity, 4 of 5 upper power segment had significantly higher LA during LV compared to LC (*p* < 0.002). No difference in LA was found when comparing the lower power segments of LV to the same segments of LC, except for the last lower LV-segments, where LA was significantly higher during LV than the corresponding LC-segments (*p* < 0.002). VP had a higher RPE on the upper power segment than the corresponding segment of CP on both intensities. On the lower power segment, there was no difference in RPE.

The mean VO_2_, LA, HR, whole-bout RPE, RER and MR during the 20 min main period of the LC, LV, HC, and HV bouts are summarized in Table 2. There was a significant main effect of both intensity and power condition on VO_2_ and LA (both *p* < 0.05, Figure 2). A significant interaction effect between intensity and power condition was found for LA (*p* < 0.01), but not VO_2_ (*p* = 0.33).

A significant main effect of intensity was found on HR, whole-bout RPE and RER (all *p* < 0.01, Figure 2). There was a tendency toward a main effect of power condition on whole-bout RPE (*p* = 0.09) as well as a tendency toward an interaction effect between intensity and power condition on whole-bout RPE and RER (*p* = 0.09 and 0.08, respectively). There was no main effect of power condition for HR or RER (both *p* > 0.24).

The mean VO_2_, LA, HR, RPE, RER, and MR during the 3 min of standardized power output prior to (PRE) and after (POST) the LC, LV, HC, and HV bouts with corresponding *p*-values and effect sizes are summarized in Table 3.

**TABLE 3:** Change scores ± standard deviation for different variables from PRE to POST for each of the four bouts.

	Δ LC	Δ LV	Δ HC	Δ HV	Cp	Cη	Ip	Iη	Intp	Intη
VO_2_ (ml/kg/min)	0.5 ± 1.0	0.3 ± 1.0	2.7 ± 1.8*	1.8 ± 1.2*	<0.01	0.505	<0.05	0.257	0.24	0.096
LA (mmol/l)	−0.4 ± 0.3*	−0.4 ± 0.6*	0.8 ± 1.0*	1.7 ± 0.8*	<0.05	0.276	<0.01	0.826	<0.01	0.408
Heart rate (bpm)	4.4 ± 2.92*	3.9 ± 4.0*	12.7 ± 6.3*	10.6 ± 7.2*	<0.01	0.721	0.24	0.099	0.43	0.044
RPE (6–20)^1^	1.5 ± 2.5*	1.7 ± 1.8*	3.1 ± 2.0*	1.9 ± 2.1*	0.06	0.237	0.16	0.139	0.06	0.221
RER (-)	−0.01 ± 0.01	−0.02 ± 0.01*	−0.02 ± 0.02*	−0.04 ± 0.02*	<0.05	0.539	<0.05	0.546	0.80	0.005
MR (W)	13.3 ± 25.2	−1.15 ± 21.2	59.6 ± 48.9*	38.8 ± 33.1*	<0.01	0.443	<0.01	0.464	0.67	0.014

Oxygen consumption (VO_2_), Blood lactate (LA), Heart rate (HR), Rating of perceived exertion (RPE), Respiratory exchange ratio (RER), Metabolic rate (MR). For VO_2_ and HR, PRE and POST are the mean of the 5th and the 28th min, respectively. For LA, PRE and POST are the mean LA measured at the end of the 5th and the 28th min, respectively. For RPE, PRE, and POST values were measured after 4.5 and 27.5 min, respectively. *P*-values and effect size (partial Eta squared) corresponding to main effects of power condition (Cp and Cη), intensity (Ip and Iη) and interaction effects (Intp and Intη). ^1^RPE refers to whole bout. *Indicates a significantly different value from PRE to POST, *p* < 0.05.

There was a main effect of intensity and power strategy on VO_2_, LA, RER, and MR from PRE to POST (*p* < 0.05). VO_2_ and MR increased more following high intensity and CP compared to low intensity and VP, respectively, whereas RER decreased more following high intensity and VP compared to low intensity and CP, respectively. LA decreased following both low intensity bouts. At the high intensity, LA increased following both bouts, and the increase was significantly bigger following VP vs. CP. There was also an interaction effect for LA with a greater effect of power condition at the high intensity compared to low intensity. There was a significant increase in HR from PRE to POST during all of the four bouts (*p* < 0.05), but the change was not significantly different between power conditions. RPE increased significantly from PRE to POST during all of the four bouts (*p* < 0.05), and the increase in RPE following HC was significantly greater than following HV (*p* < 0.05).

## Discussion

The aim of the present study was to investigate physiological and perceptual response to VP vs. CP, and to investigate if variations in power output which span above LT differ from variations below LT. The main finding was that performing the same amount of work with varying power every second minute for 20 min, had a main effect leading to a higher overall oxygen cost, heart rate and blood lactate than maintaining a constant power. At low intensity, the whole-bout RPE corresponded with the physiological parameters and were higher for the VP bout compared to CP. However, whole-bout perceived exertion was similar between the two power conditions at high intensity.

### Oxygen Cost

The finding of a higher oxygen cost during VP compared to CP at the high intensity, despite matched average work rate, is in contrast to several previous studies on the topic (Liedl et al., 1999; Palmer et al., 1999; Kang et al., 2007; Suriano et al., 2007). However, Suriano et al. (2007) used a lower load and less frequent fluctuations (i.e., 90% of LT ± 20% every 5th min) and Liedl et al. (1999) used less frequent and lower amplitude fluctuations (1-h maximal power ± 5% every 5th min).

The increases in power output during VP, both below and above the LT will require anaerobic energy contribution due to the delay in the aerobic system and will also lead to an oxygen debt (Xu and Rhodes, 1999). Acquiring an oxygen debt would lead to an underestimation of the overall oxygen cost as opposed to the increased cost seen in the present study. A greater reduction in exercise intensity will likely allow well trained cyclists to “repay” any potential oxygen debt faster. This can also be substantiated by our findings of higher VO_2_ during the first upper power segment during the HV bout compared to the corresponding period in HC. This indicates that the participants have fast VO_2_ kinetics and are able to increase their VO_2_ rapidly, thus minimizing oxygen debt, a skill useful when racing and attacking. Taken together, this might indicate that an average power output close to LT, with sufficiently large and frequent variations as in the present study may be needed to see an increased oxygen cost.

Exercising above LT also involves a slow component of VO_2_ (Lucìa et al., 2000). The VO_2_ slow component is reported to be less for professional cyclists than for less trained participants (Lucìa et al., 2000, 2002), but given the fluctuation up to 110% of LT during VP it could possibly be present. Although we found an increase in VO_2_ through the test, the increase was not different between the power conditions, and thus it does not seem that the slow component can explain the differences between the VP and CP conditions. Additionally, oxygen cost and LA were significantly higher during VP compared to CP at the low intensity. Any “unpaid” oxygen debt should also be minimal since the exercise intensity was at the most 85% of LT (Xu and Rhodes, 1999), and thus the magnitude of LA accumulation and the VO_2_ slow component should be negligible.

### Blood Lactate

As power during HV fluctuated above and below LT for a prolonged period, the finding of the present study that mean LA was higher for HV compared to HC, was not surprising, and is in agreement with most of previous studies (Palmer et al., 1999; Suriano et al., 2007; Theurel and Lepers, 2008). LA accumulation increases in an exponential manner when intensity increases (Faude et al., 2009) and thus VP with upper power segments above LT results in periods of rapid increases in blood lactate. The subsequent periods of 80% of LT results in lactate clearance, but the exponential nature means that the cyclists are able to clear less than they accumulate in the prior segment. Although the average intensity of the high intensity bouts was 95% of LT, the combination of 10 min at 110% of LT and 10 min at 80% of LT in the HV bout results in greater blood lactate accumulation compared to HC and over time. Neither Liedl et al. (1999) nor Brickley et al. (2007) found differences in mean LA between VP and CP. However, Liedl et al. (1999) used only 5% amplitude, and Brickley et al. (2007) used 30 s of 158% of critical power with 2 min recovery at 73% of critical power. A power variation amplitude of 5% may not be enough to elicit an intensity high enough for the exponential aspect of LA accumulation to take full effect, and 30 s of high power output may not be sufficient duration for LA to accumulate in significant quantity although it may be demanding in other ways.

### Metabolic Rate

The increased LA during VP, taken together with the increased oxygen cost, indicates increased energy requirements. MR was calculated to possibly get a better understanding of the energy requirements of our two power conditions. We found no increase in metabolic rate from PRE to POST during the low intensity bouts. These findings are similar to Haakonssen et al. (2013), who used a maximal and average intensity of 75% and 55% of maximal aerobic power, respectively, and is thus comparable to our “low” condition. However, our high intensity conditions produced increases in MR, and a greater increase in MR during the HC bout compared to the HV bout, due to a greater decrease in RER values.

The greater decrease in RER values after HV compared to HC indicates a greater reliance on fat metabolism. Cole et al. (2018) demonstrated that carbohydrate feeding can acutely reduce the drop in gross efficiency, and thus increase MR, during an extended cycling bout. Although our participants were able to drink between the two bouts, they could not ingest anything during the 28 min of each bout. Our trial was shorter in duration but higher in intensity, and the increase in fat metabolism indicated by RER values could possibly have produced the same result as demonstrated by Cole et al. (2018). Additionally, when including the finding that the HV bout produced more lactate than the HC bout, a difference in the anaerobic component between the bouts are present. Although it is difficult to quantify the energy equivalent from blood lactate, it will be greater than zero and thus the MR of the HV bout will underestimate the actual energy expenditure.

It should be mentioned that the VP condition ended with a low power output segment prior to the post measurement, which could potentially have led to an underestimation of VO_2_ and thus MR, due to not fully reaching steady state during the 3-min post period. However, our participants were highly competitive cyclist that are accustomed to power fluctuation and are likely to have fast VO_2_ kinetics. Additionally, there were no statistically significant difference between the two final 30 s periods of VO_2_ and RER measurements or corresponding MR calculations during the 3-min post period (*p* = 0.91, 0.74, and 0.84, respectively).

After considering potential oxygen debt, lactate accumulation and VO_2_ slow component, we are left with indications that the power fluctuations themselves both require and allows for increased oxygen consumption. Our findings indicate that power fluctuations that reach intensities above steady state differ from fluctuations within steady state.

### RPE

A similar mean RPE-score between VP and CP has been reported previously in the literature (Liedl et al., 1999; Palmer et al., 1999; Bernard et al., 2007; Kang et al., 2007; Lepers et al., 2008), but not when physiological cost differed as in the present study. Generally, it is expected that the whole-bout RPE values coincides with physiological cost, however, the power variations throughout the HV bout introduce frequent changes and this continuous alteration in the task may distract the participants from feeling increased exertion which should correspond to the observed increase in physiological cost. The whole-bout RPE does not seem to be greatly affected by the in-bout RPE which is higher during the upper power segments compared to CP (i.e., at 14.5 min) in both the high and low intensity conditions.

The variation resulting from VP could potentially influence RPE by compensating for the seated condition used in the present study. Although cycling position must be standardized because a change from seated to standing can influence physiological cost, a continuous seated position can potentially induce some local fatigue which normally could be alleviated through standing. As different power has been shown to influence both joint power (Skovereng et al., 2015) contribution and muscle activation (Hug and Dorel, 2009), the VP could potentially also alter the muscle contribution and influence the RPE.

Varying power induced higher VO_2_ and LA at both high and low intensities, but at low intensity, varying power was perceived as more exhausting, with 9 of 15 participants reporting the variable power condition to be more exhausting. To the authors’ knowledge, LIT is usually performed with CP among elite competitive cyclists. Consequently, the low intensity bouts with VP in the present study may have been somewhat unfamiliar to the cyclists compared to the high intensity VP bouts, possibly due to VP being more common in a high intensity setting such as interval sessions or racing. Given the large differences in power, comparing the findings at the low intensity in the present study to other studies is difficult. However, as LIT constitutes approximately 80% of elite cyclists total training volume and also constitutes a large portion of most road cycling mass-starts, these findings could be valuable for athletes and their coaches. As VO_2_, LA and whole-bout RPE were higher during LV than LC, it appears that the average power output obtained from stochastic low intensity periods of races (or training) cost more, both physiologically and perceptually, than if the same average power output comes from a constant endurance ride in training. However, in this regard, it should be mentioned that we obtained limited RPE measurements during the bouts and focussed on the whole-bout RPE due to practical considerations. In hindsight, additional RPE measurements would have been preferable for comparing with physiological measurements. Indeed, RPE was higher at VP during the upper power segments (i.e., 14.5 min). Considering this, a practical implication to coaches and their athletes is that the average numbers, e.g., average power and RPE, don’t necessary tell the whole story, and that it is important to add the perceived exertion behind the numbers to the equation.

### Upper and Lower Power Segments During VP

Overall, the segment differences between VP and CP were as expected and in accordance with previous reports (Liedl et al., 1999; Suriano et al., 2007), with higher and lower VO_2_ and HR values, respectively, during the upper and lower power VP-segments compared to the corresponding CP-segments. Liedl et al. (1999) did not find VO_2_ to be greater during the upper power VP-segments compared to the corresponding CP-segments, but this may be due to the average of the first and last 75 s of the segment being used for VO_2_ calculation and thus potentially underestimating the oxygen cost. Higher LA during the upper power VP-segments compared to the corresponding CP-segments are also reported by Liedl et al. (1999) and Suriano et al. (2007), but these authors reported no difference in LA during the lower power VP-segments compared to the corresponding CP-segments.

### Methodological Considerations

Due to methodological differences in frequency and amplitude of the power variations and mean exercise intensity, the results from the literature are difficult to compare. We decided on our intensity, amplitude and fluctuation frequency based on pilot testing. Our protocol allowed us to reach intensities well above LT (and thus likely above steady state) in the upper power segments of HV and to achieve a large enough difference between VP and CP in addition to sufficient time for physiological differences to be detected. Both power variation frequency and amplitude would affect the LA and VO_2_ response because of the nature of LA accumulation and VO_2_ kinetics. Hill and Gibson (2012) compared variations in power output every minute vs. every 5th min and found no effect on metabolic load. Potentially, the power variation amplitude and the intensity that power is varied around, has a greater effect on the physiological response than the frequency of the variations. Based on pilot testing, we decided that a mean intensity of 95% of LT was appropriate for the high intensity bout. With this intensity, 15% fluctuations allowed for an intensity well above LT in the upper power segments of HV (i.e., 110%). With this protocol, we also achieved a large enough difference between the VP and CP conditions. One could argue that 105% could be sufficient for exceeding steady state, but based on our pilot testing, we decided that 110% was feasible without producing too much fatigue.

The performance level of the participants is another methodological consideration that could possibly affect the results. High level cycling races differ from most other endurance sports in terms of frequent and stochastic power variations. The participants in the present study had high VO_2__max_ and PPO and were also highly competitive cyclists and thus familiar with intensity fluctuations from their experience racing high level races. Previous studies on power variation have included participants ranging from “healthy, physically active” to “highly trained” cyclists and few include information on racing experience (Palmer et al., 1997), which possibly could impact the outcome of these studies.

### Practical Implications

The VP and CP bouts of this study were designed to replicate a typical interval training session for elite competitive cyclists during their preparation period. The duration of 20 min that was chosen is also not an uncommon duration for a time trial. Despite the finding of increased oxygen cost and lactate values, RPE was similar between VP and CP at the high intensity. The cyclists were able to complete a total of 10 min at 110% of LT during HV with the same RPE-score as riding at a constant power output at 95% of LT for 20 min during the HC bout. Considering that VP may, in many cases, be a more race specific training method than CP, utilizing training with VP may be an advantageous and viable option, and we argue that the VP training sessions can be implemented in the daily training of elite cyclists. However, as we demonstrate that there is a significant difference in the physiological cost between VP and CP, coaches should be aware of this difference when designing training programs and calculating the total training load. Furthermore, as the physiological cost of work-matched VP and CP intervals with the same average power seem to differ, it could be hypothesized that when a cyclist performs intervals with spikes only above the average power, the physiological cost will increase even more.

## Conclusion

The present study show that varying power for 20 min led to a higher mean oxygen cost, heart rate and lactate than maintaining a constant power at the high intensity in a cohort of elite competitive cyclists. These findings were also evident at the low intensity. The perceived whole-bout exertion was higher for VP than CP at the low intensity, but at the high intensity, it was similar between power conditions, despite a greater in-bout RPE during the high intensity segments. Thus, training with VP seems to be a viable alternative to training with CP, at least at high intensity. Future studies should investigate physiological response to different intensities, amplitudes and possibly a degree of random power variations as seen in cycling races, in addition to longitudinal effects of training with VP vs. CP.

## Data Availability Statement

The raw data supporting the conclusions of this article will be made available by the authors, without undue reservation.

## Ethics Statement

The studies involving human participants were reviewed and approved by Norwegian Social Science Data Services. The patients/participants provided their written informed consent to participate in this study.

## Author Contributions

EK developed the research idea, contributed to the development of study design and protocol, performed the recruitment of participants, the data collection, data inspection, and the data- and statistical analyses, interpreted the results, and wrote the manuscript. KS and GE contributed to the development of study design and protocol, interpretation of the results, data- and statistical analyses, and contributed to revision of the manuscript. All authors contributed to the article and approved the submitted version.

## Conflict of Interest

The authors declare that the research was conducted in the absence of any commercial or financial relationships that could be construed as a potential conflict of interest.

## References

[B1] Atkinson G. Brunskill A. (2000). Pacing strategies during a cycling time trial with simulated headwinds and tailwinds. *Ergonomics* 43 1449–1460. 10.1080/001401300750003899 11083127

[B2] Beneke R. (2003). Methodological aspects of maximal lactate steady state-implications for performance testing. *Eur. J. Appl. Physiol.* 89 95–99. 10.1007/s00421-002-0783-1 12627312

[B3] Bernard T. Vercruyssen F. Mazure C. Gorce P. Hausswirth C. Brisswalter J. (2007). Constant versus variable-intensity during cycling: effects on subsequent running performance. *Eur. J. Appl. Physiol.* 99 103–111. 10.1007/s00421-006-0321-7 17146695

[B4] Brickley G. Green S. Jenkins D. G. Mceinery M. Wishart C. Doust J. D. (2007). Muscle metabolism during constant- and alternating-intensity exercise around critical power. *Int. J. Sports Med.* 28 300–305. 10.1055/s-2006-924354 17024627

[B5] Cole M. Hopker J. G. Wiles J. D. Coleman D. A. (2018). The effects of acute carbohydrate and caffeine feeding strategies on cycling efficiency. *J. Sports Sci.* 36 817–823. 10.1080/02640414.2017.1343956 28644716

[B6] Ebert T. R. Martin D. T. Stephens B. Withers R. T. (2006). Power output during a professional men’s road-cycling tour. *Int. J. Sports Physiol. Perform.* 1 324–335. 10.1123/ijspp.1.4.324 19124890

[B7] Faria E. W. Parker D. L. Faria I. E. (2005). The science of cycling: physiology and training - part 1. *Sports Med.* 35 285–312. 10.2165/00007256-200535040-00002 15831059

[B8] Faude O. Kindermann W. Meyer T. (2009). Lactate threshold concepts: how valid are they? *Sports Med.* 39 469–490. 10.2165/00007256-200939060-00003 19453206

[B9] Foster C. Snyder A. C. Thompson N. N. Green M. A. Foley M. Schrager M. (1993). Effect of pacing strategy on cycle time trial performance. *Med. Sci. Sports Exerc.* 25 383–388. 8455455

[B10] Haakonssen E. C. Martin D. T. Burke L. M. Jenkins D. G. (2013). Energy expenditure of constant- and variable-intensity cycling: power meter estimates. *Med. Sci. Sports Exerc.* 45 1833–1840. 10.1249/mss.0b013e31828e18e6 23470312

[B11] Hill C. F. Gibson A. (2012). The effect of power alternation frequency during cycling on metabolic load and subsequent running performance. *J. Sci. Cycling* 1 35–41.

[B12] Hug F. Dorel S. (2009). Electromyographic analysis of pedaling: a review. *J. Electromyogr. Kinesiol.* 19 182–198. 10.1016/j.jelekin.2007.10.010 18093842

[B13] Kang J. Mangine G. T. Ratamess N. A. Faigenbaum A. D. Hoffman J. R. (2007). Influence of intensity fluctuation on exercise metabolism. *Eur. J. Appl. Physiol.* 100 253–260. 10.1007/s00421-007-0424-9 17323070

[B14] Lepers R. Theurel J. Hausswirth C. Bernard T. (2008). Neuromuscular fatigue following constant versus variable-intensity endurance cycling in triathletes. *J. Sci. Med. Sport* 11 381–389. 10.1016/j.jsams.2007.03.001 17499023

[B15] Liedl M. A. Swain D. P. Branch J. D. (1999). Physiological effects of constant versus variable power during endurance cycling. *Med. Sci. Sports Exerc.* 31 1472–1477. 10527322 10.1097/00005768-199910000-00018

[B16] Lucìa A. Hoyos J. Chicharro J. L. (2000). The slow component of VO2 in professional cyclists. *Br. J. Sports Med.* 34 367–374. 10.1136/bjsm.34.5.367 11049147 PMC1756236

[B17] Lucìa A. Hoyos J. Santalla A. Pèrez M. Chicharro J. L. (2002). Kinetics of VO(2) in professional cyclists. *Med. Sci. Sports Exerc.* 34 320–325. 11828243 10.1097/00005768-200202000-00021

[B18] Palmer G. S. Borghouts L. B. Noakes T. D. Hawley J. A. (1999). Metabolic and performance responses to constant-load vs. variable-intensity exercise in trained cyclists. *J. Appl. Physiol.* 87 1186–1196. 10.1152/jappl.1999.87.3.1186 10484594

[B19] Palmer G. S. Hawley J. A. Dennis S. C. Noakes T. D. (1994). Heart rate responses during a 4-d cycle stage race. *Med. Sci. Sports Exerc.* 26 1278–1283. 7799772

[B20] Palmer G. S. Noakes T. D. Hawley J. A. (1997). Effects of steady-state versus stochastic exercise on subsequent cycling performance. *Med. Sci. Sports Exerc.* 29 684–687. 10.1097/00005768-199705000-00015 9140907

[B21] Peronnet F. Massicotte D. (1991). Table of nonprotein respiratory quotient - an update. *Can. J. Sport Sci. Rev. Can. Des Sci. Sport* 16 23–29. 1645211

[B22] Rønnestad B. R. Hansen J. (2018). A scientific approach to improve physiological capacity of an elite cyclist. *Int. J. Sports Physiol. Perform.* 13 390–393. 10.1123/ijspp.2017-0228 28657821

[B23] Seiler S. (2010). What is best practice for training intensity and duration distribution in endurance athletes? *Int. J. Sports Physiol. Perform.* 5 276–291. 10.1123/ijspp.5.3.276 20861519

[B24] Seiler S. Tønnesen E. (2009). Intervals, threshold, and long slow distance: the role of intensity and duration in endurance training. *Sport Sci.* 13 32–53.

[B25] Skovereng K. Ettema G. Van Beekvelt M. (2015). Local muscle oxygen consumption related to external and joint specific power. *Hum. Mov. Sci.* 45 161–171. 10.1016/j.humov.2015.11.009 26650852

[B26] Solli G. S. Tønnesen E. Sandbakk Ø (2017). The training characteristics of the world’s most succesful female cross-country skier. *Front. Physiol.* 8:1069. 10.3389/fphys.2017.01069 29326603 PMC5741652

[B27] Suriano R. Vercruyssen F. Bishop D. Brisswalter J. (2007). Variable power output during cycling improves subsequent treadmill run time to exhaustion. *J. Sci. Med. Sport* 10 244–251. 10.1016/j.jsams.2006.06.019 16914374

[B28] Swain D. P. (1997). A model for optimizing cycling performance by varying power on hills and in wind. *Med. Sci. Sports Exerc.* 29 1104–1108. 10.1097/00005768-199708000-00017 9268969

[B29] Sylta O. Tonnessen E. Hammarstrom D. Danielsen J. Skovereng K. Ravn T. (2016). The effect of different high-intensity periodization models on endurance adaptations. *Med. Sci. Sports Exerc.* 48 2165–2174. 10.1249/mss.0000000000001007 27300278

[B30] Theurel J. Lepers R. (2008). Neuromuscular fatigue is greater following highly variable versus constant intensity endurance cycling. *Eur. J. Appl. Physiol.* 103 461–468. 10.1007/s00421-008-0738-2 18415118

[B31] Wells M. S. Marwood S. (2016). Effects of power variation on cycle performance during simulated hilly time-trials. *Eur. J. Sport Sci.* 16 912–918. 10.1080/17461391.2016.1156162 26949050

[B32] Xu F. Rhodes E. C. (1999). Oxygen uptake kinetics during exercise. *Sports Med.* 27 313–327. 10.2165/00007256-199927050-00003 10368878

